# Catching global interactions in vivo

**DOI:** 10.1186/s13578-017-0177-z

**Published:** 2017-09-29

**Authors:** Yi Qiu, Suming Huang

**Affiliations:** 1 Department of Anatomy and Cell Biology, University of Florida College of Medicine, Gainesville, FL 32610 USA; 2 Department of Biochemistry & Molecular Biology, University of Florida College of Medicine, Gainesville, FL 32610 USA; 3 UF Health Cancer Center, University of Florida College of Medicine, Gainesville, FL 32610 USA; 4 Macau Institute for Applied Research in Medicine and Health, State Key Laboratory of Quality Research in Chinese Medicine, Macau University of Science and Technology, Avenida Wai Long, Taipa, Macau, China

**Keywords:** bPPI-seq, Chromatin, H2A.Z, H2A.Z interacting proteins, Transcriptional regulation

## Abstract

Histone proteins and transcription factors (TFs) play critical roles in gene transcription and development of multicellular organisms. Although antibody mediated protein isolation couple with mass spectrometry approach has been a standard method to identify TF interacting partners and characterize their functional molecular complexes, it becomes urge to develop a robust method to functional characterize how these transcription factors act during biological process in the post-human genome project era. Here, Dr. Zhao and his colleagues in the National Heart, Lung, and Blood Institute of NIH develop a sensitive and robust strategy to globally identify and characterize in vivo protein–protein interactions termed bait protein–protein interaction-sequencing (bPPI-seq) (Zhang et al. in Cell Res doi:10.1038/cr.2017.112, 2017). As a proof-of-principle, they demonstrated that genome-wide interacting partners of histone variant H2A.Z are mainly involved in transcriptional regulation which is distinct from the interacting proteins of canonical histone H2A. Thus, their results suggest that bPPI-seq can be widely used to globally characterize protein complexes especially transcription factor interacting partners and molecular networks formed.

Transcription is a key mechanism underlying the control of gene activities and cell identity during animal development and disease. Expression of genes underlying cell fate choices is coordinated by the binding of lineage-specific transcription factors to gene-proximal promoters or distal enhancers. As increased number of these pioneer and lineage specific key transcription factors have been isolated and identified, the underlying molecular mechanisms by which these factors establish regulatory networks to act on enhancers and promoters for gene expression remain poorly understood. One important way to understand the molecular mechanisms of these TFs in establishing their function is to identify TF interacting partners and to characterize protein co-regulatory complexes that these factors form [2, 3]. Traditionally, antibody mediated affinity purification couple with mass spectrometry strategies have been employed for this propose [4]. However, this method suffers several limitations that affect reproducibility of purification approaches. First, the traditional protein isolation requires large volumes of starting materials and it may not be feasible for studies involved in certain cell lineages or disease samples. Second, the most of TF and co-regulator interactions are dynamics and depended on cellular differentiation stage. Weak interactions may not be detected using the traditional method. Third, as a transcription factor, their interactions required a physiological chromatin environment. The in vitro protein purification will not resemble in vivo environment, therefore, many in vivo interactions are not able to be detected using the traditional affinity purification. Finally, the antibody pull down mediated affinity purification depends on binding affinity and quality of antibody which may result in variable results of purifications. It becomes urge to develop a robust method to access and to functional characterize these transcription factor complexes during biological process in the post-human genome project era.

Recently, a team of researchers led by Dr. Keji Zhao of the National Heart, Lung, and Blood Institute, National Institute of Health, has developed a novel strategy to identify protein interacting partners in a genome-wide scale [1]. The method termed bait protein–protein interaction-sequencing (bPPI-seq) takes advantage of the fact that active green fluorescent protein (GFP) can be reconstituted and emit fluorescent light from two half GFP moieties when they are brought to a close proximity through protein–protein interaction [5]. In bPPI-seq, the bait protein is fused to N-terminal GFP moiety while whole genome endogenous genes are randomly tagged by exon trapping using an enhanced retroviral mutagen vector containing cDNA of C-terminal half of GFP moiety and a doxycycline-inducible promoter driven splicing donor to induce a hybrid transcript of GFPC and in frame fused endogenous genes that allow expression of GFPC fusion proteins. When bait interacts with the endogenous tagged polypeptides, the cells will emit GFP light and become green cells. The hybrid RNA transcripts encoding bait interacting partners are then purified, reverse transcribed, and amplified with specific ligated primers for construction of RNA-seq library. Finally, the libraries are subjected to next generation genome-wide sequencing to identified protein bait interacting partners within cells. This is a robust and sensitive genome-wide strategy to interrogate transcription factor and cofactor function and their regulatory networks. One example is the study of distinct molecular functions of canonical histone H2A and H2A.Z variant.

Chromatin plays critical role in transcription by assembling genome into nucleosomes and modulating accessibility of transcription regulators and RNA polymerases. Whereas histone H2A is core structure component of nucleosome mainly involved in packaging DNA into chromatin during genome replication, H2A.Z replaces core H2A in promoter regions of genes during transcription cycle. H2A.Z containing nucleosomes facilitate promoter DNA accessibility and transcription activation [6, 7]. Thus, what is molecular mechanism underlying functional difference between H2A and H2A.Z? By employing bPPI-seq strategy, Dr. Zhao group identified two distinct sets of interacting partners involved in complete different biological processes for H2A and H2A.Z. Variant H2A.Z interacting partners include transcription factors, histone chaperones, and chromatin remodeling complexes that are critical for gene transcription and regulation [1]. These H2A.Z specific interacting proteins were further validated using traditional co-immunoprecipitation and ChIP-seq analysis [1]. Thus, Dr. Zhao and his colleagues described a novel experimental strategy allowing characterization of TF complex composition and their relevant function in vivo.

In summary, the novel bPPI-seq provides a strategy for genome-wide identification of protein–protein interaction network in physiological condition and can be widely applied to characterize the molecular complexes by which transcription factor forms and establishes function.
